# Fecal Calprotectin Excretion in Preterm Infants during the Neonatal Period

**DOI:** 10.1371/journal.pone.0011083

**Published:** 2010-06-11

**Authors:** Carole Rougé, Marie-José Butel, Hugues Piloquet, Laurent Ferraris, Arnaud Legrand, Michel Vodovar, Marcel Voyer, Marie-France de la Cochetière, Dominique Darmaun, Jean-Christophe Rozé

**Affiliations:** 1 INRA UMR 1280, Physiologie des Adaptations Nutritionnelles, Université de Nantes, CRNH, Nantes, IMAD, CHU de Nantes, Nantes, France; 2 EA 4065, Ecosystème Intestinal, Probiotiques, Antibiotiques, Faculté des Sciences Pharmaceutiques et Biologiques, Université Paris Descartes, Paris, France; 3 INSERM, CIC 0004, Neonatal Intensive Care Unit, Hôpital Mère-et-Enfant, CHU de Nantes, Nantes, France; 4 Neonatal Intensive Care Unit, Institut de Puériculture, Paris, France; 5 EA 3826, Thérapeutiques Cliniques et Expérimentales des Maladies Infectieuses, Nantes, France; Columbia University, United States of America

## Abstract

**Background:**

Fecal calprotectin has been proposed as a non-invasive marker of intestinal inflammation in inflammatory bowel disease in adults and children. Fecal calprotectin levels have been reported to be much higher in both healthy full-term and preterm infants than in children and adults.

**Objective:**

To determine the time course of fecal calprotectin (f-calprotectin) excretion in preterm infants from birth until hospital discharge and to identify factors influencing f-calprotectin levels in the first weeks of life, including bacterial establishment in the gut.

**Methodology:**

F-calprotectin was determined using an ELISA assay in 147 samples obtained prospectively from 47 preterm infants (gestational age, and birth-weight interquartiles 27–29 weeks, and 880–1320 g, respectively) at birth, and at 2-week intervals until hospital discharge.

**Principal Findings:**

Although median f-calprotectin excretion was 138 µg/g, a wide range of inter- and intra-individual variation in f-calprotectin values (from day 3 to day 78) was observed (86% and 67%, respectively). In multivariate regression analysis, f-calprotectin correlated negatively with ante and per natal antibiotic treatment (p = 0.001), and correlated positively with the volume of enteral feeding (mL/kg/d) (p = 0.009), the need to interrupt enteral feeding (p = 0.001), and prominent gastrointestinal colonization by *Clostridium sp* (p = 0.019) and *Staphylococcus sp* (p = 0.047).

**Conclusion:**

During the first weeks of life, the high f-calprotectin values observed in preterm infants could be linked to the gut bacterial establishment.

## Introduction

Initially called leukocyte L1, calprotectin is a 36 kDa calcium and zinc binding protein that constitutes about 60% of soluble cytosol protein in human neutrophil granulocytes, and is found in monocytes, macrophages and epithelial cells [1]. Calprotectin is thought to regulate inflammatory processes [2], and exert antimicrobial and anti-proliferative properties *in vitro* [3], [4] and *in vivo* [5]. Its resistance to proteolysis and stability even after a week of storage at room temperature facilitate its determination in faeces [6].

High fecal calprotectin (f-calprotectin) levels were shown to correlate with an increased turnover of leukocytes in the intestinal barrier and granulocyte migration towards intestinal lumen [7], [8]. Hence, f-calprotectin has been proposed as a non-invasive marker of intestinal inflammation in inflammatory bowel disease in adults [9] and children [10]. A recent review points the relevance and limitations of calprotectin determination in these clinical settings [11].

F-calprotectin levels have been reported to be much higher during the first few weeks of life both in healthy full-term [12]–[15] and preterm infants [12], [16]–[19] than in healthy adults [6] and children [13], [14], [20]. Despite these high levels, the putative use of f-calprotectin as a marker of gastrointestinal disease, particularly necrotizing enterocolitis (NEC), has been explored in several cohorts of preterm infants. However, due to the high inter- and intra-individual variations consistently observed by all authors the determination of a cutoff value for f-calprotectin has remained an elusive goal, as cut-off values ranging from 200 to 2000 µg/g have been proposed [16], [18], [21], [22]. Although earlier studies have assessed calprotectin concentrations in preterm infants, the factors that affect its excretion in neonates are incompletely known and remain controversial [12], [16], [17], [19], [22], [23]. Earlier studies suggest f-calprotectin is higher in infants born by cesarean section, compared with vaginal delivery, and correlates positively with postnatal age and volume of enteral feeds, and negatively with antibiotic treatment. The latter factors are known to influence gut bacterial colonization, suggesting a possible relationship between bacterial establishment and calprotectin levels in neonatal period. Accordingly, Mohan et al found that bifidobacterial supplementation was associated with a significant decrease in calprotectin level [24].

In this context, it is of interest to improve our understanding of factors influencing the f-calprotectin excretion in preterm infants. The aims of the current study therefore were to describe the time course of f-calprotectin excretion in preterm infants from birth through hospital discharge, and to identify factors influencing its fecal excretion during the first weeks of life, including bacterial establishment in the gut.

## Materials and Methods

### Patients

Written, informed parental consent was obtained prior to inclusion, according to protocols approved by the local Institutional Review Board (Comité de Protection des Personnes dans la Recherche Biomédicale des Pays-de-la-Loire, Nantes, France). Preterm infants enrolled in the current study were part of a larger controlled trial on probiotic supplementation [25]. Preterm infants admitted to the neonatal intensive care units at the Mère-Enfant Hospital (Nantes, France) and at the Institut de Puériculture (Paris, France) were eligible for enrolment in the prospective study if they met the following inclusion criteria: a gestational age <32 weeks, a birth weight <1500 g, a postnatal age no greater than 2 weeks, and the absence of any disease other than those linked to prematurity. Infants were fed with human milk and/or with a preterm formula. They were randomly assigned to receive from the start of enteral feeding until discharge from the NICU four daily capsules of a supplement containing either (a) maltodextrin alone (referred to as placebo group), or (b) 10^8^ lyophilized cells per unit of *Lactobacillus rhamnosus* GG (Valio, Ltd., USA) and *Bifidobacterium longum* BB536 (Morinaga Milk Industry Co., Ltd., Japan) and maltodextrin (referred to as probiotic group).

### Clinical data

Collected clinical parameters included gestational age, birth weight, sex, mode of delivery (vaginal or cesarean section), intrauterine growth retardation, 5-min Apgar score, maternal treatment (corticoids and antibiotics). In addition, postnatal age, type of enteral feeding (own mother's milk, bank milk, or preterm formula), postnatal treatment (antibiotics), volume of enteral feeding (mL/kg/d), and number of enteral feeding interruptions were recorded before each stool sample was collected. Poor tolerance to enteral feeding was defined as a suspension of enteral feeding for any day during the week following the day the fecal sample had been obtained. After clinical assessment, enteral feeding was interrupted if there were significant residues in gastric aspirates, abdominal distension, and/or blood in stool. As enteral feeding is routinely suspended when ibuprofen is administered (to promote the closure of *ductus arteriosus*), the planned, on purpose interruptions of enteral feeding motivated by the prescription of ibuprofen treatment, were not considered as an event of poor GI tolerance.

### Stool collection

Stool collection was performed on the first 24 infants enrolled in each neonatology unit for the follow-up of fecal microbiota and fecal calprotectin. Stool samples were collected weekly from diapers in two sterile plastic tubes until hospital discharge. One of the tubes contained 0.5 mL of brain heart infusion (BHI) with 15% glycerol as a cryoprotective agent for microbiota analysis. All fecal samples were frozen and stored at -80°C immediately after collection until analysis.

### Microbiological analysis of fecal samples

Fecal microbiota was analyzed weekly. After thawing the samples collected in BHI, serial dilutions were performed, and spread using the automatic spiral system (Chemunex-AES Laboratoire, Bruz, France) on various media allowing the isolation of the main genera found in preterm infants' fecal microbiota, i.e. staphylococci, enterococci, enterobacteria, lactobacilli, clostridia, *Bacteroides*, and bifidobacteria, as previously described [26]. Bacterial counts were expressed as the log_10_ CFU/g of feces and the count threshold was 3 log_10_ CFU/g of feces. Bacterial identification was performed at the species level using routine laboratory methods and Wadsworth laboratory procedures for anaerobes [27]. Moreover, for bifidobacteria, identification of genus *Bifidobacterium* was performed by genus PCR [28], and species determination using 16S rRNA gene sequencing. The 2 probiotic strains were detected specifically by a culture-polymerase chain reaction method.

### Fecal calprotectin determination

Before analysis, frozen stool samples were thawed at room temperature. Fecal calprotectin concentrations were determined in duplicate at 2-week intervals, using a commercial enzyme linked immunoassay (Calprest®, Eurospital, Trieste, Italy). The mean coefficient of variation was less than 3%.

### Statistical analysis

Statistical analysis was performed using SPSS® version 14.0 (SPSS, Chicago, IL). Results are reported as median and interquartiles, and percentages were presented as percent [95% confidence interval]. Chi2 and Mann-Whitney U test were used to compare proportion and quantitative values respectively. Spearman's correlation test was used to evaluate the relationship between selected perinatal or neonatal characteristics and fecal calprotectin values. All variables that showed a statistically significant correlation with fecal calprotectin by univariate analysis were subsequently analyzed together in forward and backward stepwise, multivariate regression analyses, after log transformation of fecal calprotectin values. Statistically significant differences were assumed when p<0.05.

## Results

### Patients

Forty-seven preterm infants (29 boys and 18 girls) were enrolled in the current study. They had a median gestational age of 29 weeks [interquartile, 27–29 weeks], a median birth-weight of 1100 g [interquartile, 880–1320 g]. Sixteen were born by caesarean section and 31 by vaginal delivery. Median Apgar score at 5 minutes was 10 [interquartile, 8–10]. During follow-up, 17 infants were fed solely with human milk, 29 with human milk combined with a preterm infant formula, and 1 with a preterm infant formula. As this study was part of a larger clinical trial on probiotic supplementation, 25 of the 47 infants received a milk supplemented with the probiotic strains from the start of enteral feeding (median: 2 days of life, [interquartile, 2–4]) until discharge from hospital (median: 62.5 days of life, [interquartile, 45.5–78.5]). The median time [interquartile] to full enteral feeding (100% of total calories supplied via the enteral route) was 20.0 days [interquartile, 14.5–30.0]. Twenty-five of the 47 infants were born from mothers who received antibiotics (12 during the two last month of pregnancy, 7 during delivery and 6 during both), 39 received antibiotics due to a materno-fetal infection (median duration: 2 days, [interquatile, 2–3]) and 25 received antibiotics due to a nosocomial infection (mean [SD] cures: 2.7 [0.8]; median cumulated duration: 15.1 days, [interquartile: 9–20]). The antibiotics included broad-spectrum cephalosporins, penicillins ± β-lactamase inhibitors, glycopeptides, and/or aminosides used either alone or in combination.

### Gut bacterial establishment

The data from culture-based analyses showed that gastrointestinal tract of all infants was poorly colonized, harbouring no more than seven bacterial species (Table 1). The most common bacteria isolated were staphylococci (Table 1). In contrast, colonization by enterococci occurred in only one third of infants at a low level and enterobacteria were isolated in 23 infants. With regard to anaerobes, *Bacteroides* was isolated in only one infant whereas clostridia were isolated in one half of infants. Lactobacilli and bifidobacteria, which were identified as the two probiotic strains were found in 26 and 21 infants, respectively.

**Table 1:** Bacterial colonization in enrolled preterm infants from week 1 to week 6.

	Weeks 1–2 (n = 43)^a^		Weeks 3–4 (n = 39)		Weeks 5–6 (n = 22)	
**Aerobic genera**						
Staphylococci	41 (95%)^b^	7.4 (3.3–8.8)^c^	39 (100%)	7.3 (3.8–9.5)	21 (95%)	6.9 (3.8–8.2)
Enterococci	5 (12%)	5.3 (3.3–8.8)	11 (28%)	6.1 (3.9–9.0)	9 (41%)	6.5 (4.7–10.1)
Enterobacteria	14 (33%)	8.4 (3.3–9.5)	21 (54%)	8.8 (3.3–10.0)	13 (59%)	8.5 (5.0–9.2)
Lactobacilli	23 (53%)	7.4 (3.3–9.0)	26 (67%)	7.1 (3.6–9.4)	14 (64%)	6.1 (3.6–8.7)
**Anaerobic genera**						
*Bacteroides*	1 (2%)	8.4	1 (3%)	6.1	1 (5%)	6.6
*Clostridium*	11 (26%)	5.9(3.3–9.0)	22 (56%)	6.9 (3.3–8.2)	13 (59%)	6.0 (4.1–7.5)
Bifidobacteria	16 (37%)	6.2 (3.3–9.1)	14 (36%)	5.7 (3.3–10.0)	9 (41%)	4.8 (3.3–8.4)

a number of analyzed infants.

b number of colonized infants (%).

c bacterial counts expressed as log_10_ CFU/g of feces (median, range) in colonized infants, threshold  = 3.0.

### Fecal calprotectin concentrations

Throughout follow-up (from day 3 to day 78), a total number of 147 fecal samples were obtained, with an average of 3 samples per infant. The median fecal calprotectin level calculated on all 147 samples was 138 µg/g of feces [interquartile, 58–271 µg/g], ranging between 15 and 811 µg/g. A wide range of calprotectin levels were observed, with inter- and intra-individual coefficients of variation of 86% and 67%, respectively. Fecal calprotectin levels were not significantly different in the placebo and probiotic groups throughout the study (p = 0.31) [25]. There was no effect of center, as median calprotectin levels were 180 µg/g [interquartile 52–283], and 117 µg/g [interquartile 65–234] in centers 1 and 2, respectively (NS).

The first initial fecal samples were obtained at 11 days of life (n = 47, [interquartile, 7–15 days of life]) and the median fecal calprotectin levels were 87 µg/g [interquartile, 35–153 µg/g]. In these fecal samples, fecal calprotectin levels was not affected by gender (p = 0.39), nor the use of corticoids *ante partum* (p = 0.66), or the mode of delivery (p = 0.37). In contrast, fecal calprotectin levels were significantly lower in infants whose mothers had received antibiotics *ante* and *per partum* (n = 25), compared with infants who did not receive antenatal antibiotics (n = 22)(median: 58 µg/g, [interquartile, 31–106 µg/g *vs.* median: 132 µg/g, [interquartile, 86–227 µg/g]; p = 0.005). Otherwise, there was no statistically significant correlation between fecal calprotectin levels and birth-weight (r = 0.08, p = 0.59), nor gestational age (r = 0.09, p = 0.55), or intrauterine growth evaluated by birth weight Z-score (r = 0.16, p = 0.29).

**Figure 1:**
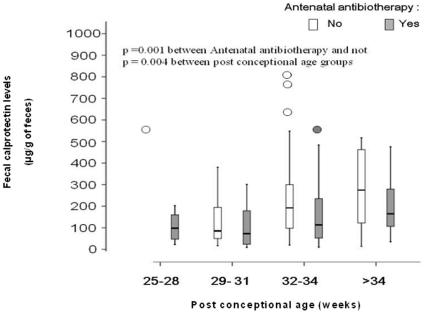
Fecal calprotectin levels in preterm infants with or without antenatal antibiotherapy. The boxplot shows the median (central horizontal line) and includes the 25^th^ (lower box border) to 75^th^ percentile (upper box border) of fecal calprotectin (µg/g of feces) in preterm infants with (74 fecal samples) and without (73 fecal samples) antenatal antibiotherapy following the postconceptional age (weeks).

The second set of samples were obtained in 43 infants at a median age of 23 days [interquartile, 20–28 days of life], the third set of sample were obtained in 22 infants at a median age of 37 days [interquartile, 34–42 days of life], and the fourth one from 9 infants at a median age of 56 days [interquartile, 46–65 days of life]. When considering the entire set of 147 fecal samples obtained in univariate analysis, fecal calprotectin levels were significantly lower in infants whose mothers had received antibiotics *ante* and *per partum* (n = 74), compared with infants who had not received any antenatal antibiotic treatment (n = 73)(median: 94 µg/g, [interquartile, 41–225 µg/g] *vs.* median: 183 µg/g, [interquartile, 94–320 µg/g]; p = 0.002) (Fig 1). Likewise, fecal calprotectin levels were significantly lower in infants with postnatal antibiotherapy (n = 35), compared with infants without postnatal antibiotherapy (n = 112)(median: 97 µg/g, [interquartile, 32–206 µg/g] *vs.* median: 155 µg/g, [interquartile, 77–298 µg/g]; p = 0.001). Otherwise, fecal calprotectin levels found in infants who had received enteral feeding the day prior to sampling (n = 125) were not significantly different from those who had not been fed enterally the day before (n = 22)(median: 147 µg/g, [interquartile, 71–268 µg/g] *vs.* median: 113 µg/g, [interquartile, 32–283 µg/g]; p = 0.50).

**Figure 2:**
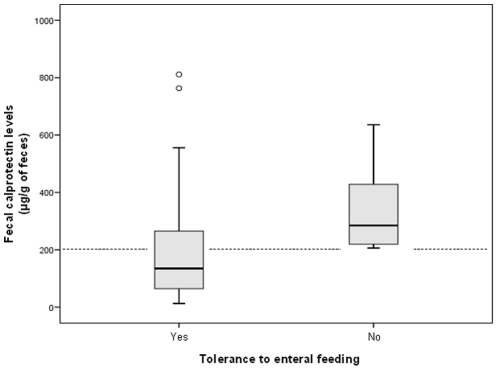
Fecal calprotectin levels in preterm infants without or with poor tolerance to enteral feeding. Fecal calprotectin in preterm infants without poor tolerance to enteral feeding (i.e. with unplanned enteral feeding interruption, n = 118), and with poor tolerance to enteral feeding (i.e. with unplanned enteral feeding interruption, n = 7). The boxplot shows the median (central horizontal line) and includes the 25^th^ (lower box border) to 75^th^ percentile (upper box border). Dotted line represents cut-off level (205 µg/g of feces) for poor tolerance to enteral feeding (see text for details).

Among infants receiving enteral feeding the day before sampling, fecal calprotectin levels were significantly higher in infants who received formulas as their exclusive or predominant source of feeding (n = 21) than in those fed human milk (n = 104) (median: 226 µg/g [interquartile, 116–334 µg/g] *vs.* median: 126 µg/g [interquartile, 55–237 µg/g]; p = 0.02), and were significantly higher in infants with poor tolerance to enteral feeding (n = 7) than infants with good tolerance to enteral feeding (n = 118) (median: 285 µg/g, [interquartile, 208–552 µg/g] *vs.* median: 132 µg/g, [interquartile, 62–260 µg/g]; p = 0.008) (Fig 2). By the calculation of the receiver operator curve (ROC), a cut-off level of 205 µg/g of feces was defined for the detection of poor tolerance in preterm infants and area under the ROC curve was 0.80±0.06 (p = 0.08). A fecal calprotectin levels more than 200 µg/g has a good sensibility of 1.0 [0.72–1.0] but a low specificity of 0.64 [0.57–0.71] to predict poor tolerance to enteral feeding.

Among the 147 fecal samples analyzed, fecal calprotectin levels were positively correlated with the volume of enteral feeding (mL/Kg/day) (r = +0.36, p = 0.01), postnatal age (r = +0.17, p = 0.036) and postconceptional age (r = +0.34, p = 0.001), and negatively correlated with weight gain (expressed in weight Z-score gain)(r = −0.19, p = 0.03).

In the samples analyzed by culture methods (n = 128), there were significant positive correlations between fecal calprotectin levels and intestinal colonization levels by *Staphylococcus* (r = +0.18, p = 0.04), *Enterococcus* (r = +0.19, p = 0.03) and *Clostridium* (r = +0.22, p = 0.011). In contrast, no significant correlations were found between fecal calprotectin levels and enterobacteria (r = +0.12, p = 0.15), nor *Bifidobacterium* (r = +0.11, p = 0.19). Nevertheless, fecal calprotectin levels and *Lactobacillus* tended to be positively correlated (r = +0.17, p = 0.06).

In multivariate regression analysis, fecal calprotectin correlated negatively with ante and per natal antibiotic treatment (p = 0.001), and correlated positively with the volume of enteral feeding (mL/kg/d) (p = 0.009), the need to interrupt enteral feeding (p = 0.001), and prominent gastrointestinal colonization by *Clostridium sp* (p = 0.019) and *Staphylococcus sp* (p = 0.047) (Table 2).

**Table 2:** Main explanatory variables found by multivariate analysis for fecal calprotectin values.

Variables	Non standardized coefficient	Standardized coefficient	p-value
(R^2^ = 0.32, n = 125)			
Ante/per natal antibiotic treatment (yes/no)	−0.558	−0.276	0.001
Volume of enteral feeding (mL/Kg/d)	+0.004	+0.236	0.009
Unplanned interruptions of enteral feeding (yes/no)	+0.920	+0.257	0.001
*Clostridium* (>10^5^ CFU/g of feces) (yes/no)	+0.468	+0.206	0.019
*Staphylococcus* (>10^5^ CFU/g of feces) (yes/no)	+0.347	+0.206	0.047

## Discussion

The findings of the current study confirm that fecal calprotectin levels are elevated in premature infants, compared with older age groups, with a wide range of inter- and intra-individual variation during the first few weeks of life. They further demonstrate that the most significant factors that affect calprotectin excretion are ante and per natal antibiotic treatment, volume of enteral feeding (ml/Kg/day), the occurrence of unplanned interruptions of enteral feeding, and the gastrointestinal bacterial colonization.

Consistent with previous studies [12], [16]–[18], the fecal calprotectin levels observed in healthy preterm infants in the current study were high and clearly exceed those reported in healthy adults and children. Likewise, we observed a wide range of inter- and intra-individual variation. Such range of variation is unlikely to result from a poor stability of calprotectin in stools, since calprotectin resists proteolysis, and is stable at room temperature for up to a week [29]. Although stool samples were collected from babies' diapers in the current study (as in all studies carried out in infants), the sampling technique cannot account for the variation either, since the changes in calprotectin concentrations found in the current study largely exceed what could be accounted for by water absorption [14]. Besides, the changes observed cannot arise from the ingestion of calprotectin present in human milk, since, in agreement with earlier findings [14], none of the human milk samples we analyzed (n = 10, data not shown) contained any detectable calprotectin (threshold: 15 mg/L). Thus, the wide range of f-calprotectin values may reflect true inter- and intra-individual variability in calprotectin fecal excretion in that patient population.

The high calprotectin levels observed in neonates may reflect the increased transepithelial migration of neutrophil granulocytes and/or macrophages into the intestinal lumen of preterm infants. As Berstad et al reported a significant correlation between calprotectin levels in gut lavage fluid and intestinal permeability [8], the increased migration of neutrophil granulocytes and/or macrophages into the gut lumen might be related to the higher intestinal permeability associated with intestinal mucosal immaturity. However, f-calprotectin levels are similar in preterm and full term infants, although intestinal permeability is higher in preterm than in term infants [30].

In univariate analysis, levels of calprotectin in the initial samples did not correlate with gestational age, birth weight or the mode of delivery, supporting earlier reports [16], [17]. Although other authors found a correlation of calprotectin with gestational age, the latter correlation was found with early determination in meconium, whereas calprotectin levels subsequently decreased during the first week postnatal [16], [19]. Likewise, we found no significant correlation between f-calprotectin and type of feeding (formulas as exclusive or predominant source of feeding *vs.* human milk) in univariate analysis, consistent with other studies [16]–[18].

By contrast, gut microbiota appeared to influence calprotectin excretion in the present cohort, as suggested by Josefsson et al [16]. In the current study gut microbiota was analyzed by culture, which allowed the isolation and identification of the main bacterial genera, even in a sub-dominant status. A limitation of this approach could be the inability to detect the uncultivable part of the microbiota. However, by contrast with adult gut microbiota, the latter accounts for a very small fraction of the overall bacterial population in preterm neonates [31], [32]. We found correlations between f-calprotectin and both microbiota *per se*, and other factors known to influence gut bacterial establishment as well. The current report thus is first to provide evidence for an effect of intestinal bacterial colonization on fecal calprotectin excretion Three lines of evidence point to such effect. First in both univariate and multivariate analyses, f-calprotectin correlated positively with intestinal colonization by *Staphylococcus* and *Clostridium*. Secondly, in univariate analysis, fecal calprotectin levels correlated positively with postnatal and postconceptional ages, factors known to influence the gut microbiota composition. However, this correlation did not remain significant in multivariate analysis in accordance with Campeotto et al [17], as opposed to the study by Josefsson et al [16]. Thirdly, the use of antibiotics impacted f-calprotectin levels. Indeed, throughout the study f-calprotectin correlated negatively with ante and per natal antibiotics in univariate and multivariate analyses. To our knowledge, the current study is first to demonstrate such impact. Indeed, earlier studies have shown changes in the gut microbiota establishment in infants born from mothers who had received antibiotic *per partum* [33]. Likewise, a negative correlation was found with neonatal antibiotic courses in univariate analysis, but in contrast to other studies [16] this correlation did not remain significant in multivariate analysis. However, in the latter study a correlation was only observed in infants treated with cefotaxim and meropenem, two broad-spectrum antibiotics.

To summarize, factors known to delay gut bacterial colonization (both ante- and post-natal antibiotic treatments) correlated negatively with fecal calprotectin levels, whereas factors known to favor gut bacterial colonization (gestational age and postconceptional age) correlated positively with fecal calprotectin levels. This is in accordance with the study of Mohan et al, who described a decrease in f-calprotectin levels in infants supplemented with a probiotic strain [24]. In the latter study, probiotic supplementation increased bifidobacteria levels, and decreased the levels of clostridia, a genus positively correlated in with f-calprotectin in the current study. By contrast, a recent study did not find any correlation between gut microbiota colonization and f-calprotectin: however, the culture techniques used in that report did not allow detection of clostridia [23].

Otherwise, we observed a highly significant, positive correlation between the volume of enteral feeding (mL/Kg/day) and f-calprotectin excretion in multivariate analysis, as previously reported [16].

Taken together, the current results suggest that exposure to two kinds of luminal ‘antigens’−i.e, commensal intestinal bacteria, and dietary antigens− might induce a state of “physiological” subclinical intestinal inflammation in preterm infants as well as in full-term infants. It is tempting to speculate that intestinal bacteria, and specific, individual components of the commensal microbiota might have variable abilities to stimulate transepithelial granulocyte migration and/or to induce calprotectin release from leucocytes and macrophages, as shown in gnotobiotic piglets colonized with various strains of *Escherichia coli* [34]. Thus, the stress induced by birth *per se* and by the adaptation to the extra-uterine life, particularly concerning gut bacterial colonization, rather than the degree of mucosal maturation or term of birth, may account for the higher fecal calprotectin levels found in infants, compared to older age groups. This high intestinal expression of calprotectin, known to display antimicrobial properties, might participate in the mechanisms of defense in neonates, whose intestinal immune system is not mature.

Despite these “physiological” high levels of f-calprotectin, several studies strongly suggest that a rise in f-calprotectin above this high, baseline levels may be a candidate, non-invasive marker of gastrointestinal diseases, in particular NEC [16]–[18], [21], [22]. These studies reported a significant rise in f-calprotectin levels in infants suffering from gastrointestinal disease, particularly from NEC. Several thresholds for suspicion of NEC have been proposed, i.e. 200mg/L in Caroll's study [22], 2000 µg/g in the study by Josefsson [16], and 636 µg/g for the study by Campeotto et al [21]. The threshold in Caroll's study appears too low. Indeed, the latter study, performed in a very small group of infants (6 infants with NEC, and 6 healthy controls), reported low f-calprotectin values in healthy preterm infants as compared with the literature. In our study, we did not observe any case of NEC, and median f-calprotectin excretion was 138 µg/g [interquartile, 58–271 µg/g]. Using the three thresholds reported among the infants in our cohort 34 infants should have been suspected of having NEC using Caroll's threshold, 0 with Josefsson's cut-off, and 3 with Campeotto's threshold. The rise in calprotectin levels we observed in cases of intolerance to enteral feeding suggests the monitoring of f-calprotectin might be useful as a warning signal for gastrointestinal disease and/or poor tolerance of enteral nutrition. However, the lack of specificity we observed might be due to the “physiological inflammation” linked to the bacterial colonization. Many more studies with much larger cohorts are warranted to confirm whether such thresholds or cut-off values could be recommended for routine use in clinical practice.

**Figure 3:**
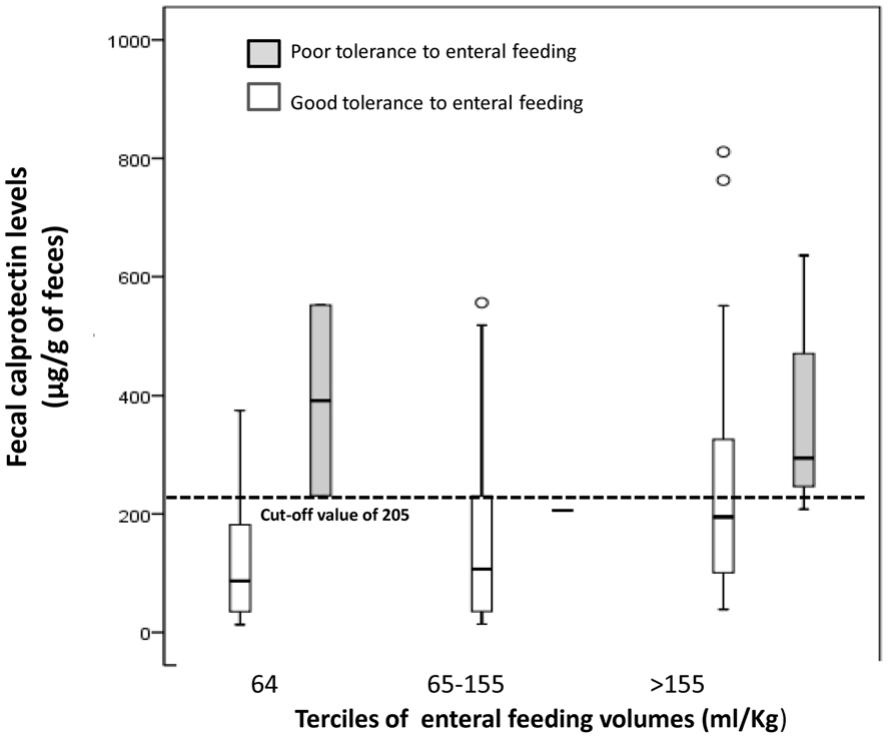
Fecal calprotectin as a function of the tercile of enteral feeding volume and tolerance to enteral feeding. The median and interquartile range of fecal calprotectin are plotted for each volume of enteral feeding in infants with good (open boxes) or poor (closed boxes) tolerance to feeding. Poor tolerance to feeding was defined as the need for unplanned enteral feeding interruption.

Finally, it may seem paradoxical to observe that a rise in calprotectin is observed in parallel with increased volume of enteral feeding, but a rise is also observed when there is a need to interrupt enteral feeding, i.e., in cases of poor digestive tolerance. To further address this issue we divided samples according to the terciles of enteral feeding volume received, and, within each tercile, we separated samples depending on the need to interrupt enteral feeding or not. As shown in Fig 3, on one hand, calprotectin tends to be higher for the upper tercile of enteral feeding volume: this is consistent with the fact that calprotectin increases with enteral volume. On the other hand, for any given tercile of enteral volume administered, calprotectin was higher when feeding intolerance occurred. Should we have very large numbers of feeding interruptions within each tercile, we might be able to define ‘safe’ levels of calprotectin for a given volume of feeding. Interestingly, all instances when feeding had to be interrupted were associated with a calprotectin level >205 µg/g. This is consistent with the good sensitivity of calprotectin, yet this cutoff level has a poor specificity as well, as discussed with the ROC curve (see above).

In conclusion the current study demonstrates for the first time that calprotectin excretion can be linked to the gut bacterial establishment. We speculate that the enhanced expression of this protein possessing many potential functions including antimicrobial properties may participate in the innate immune system, and thus be of benefit for the developing gut in both full term and premature infants. Clinical situations may therefore occur when a pathological rise in calprotectin might be offset by a physiological increase.
